# Polyphasic characterization of *Nocardioides aquaegermanicae* sp. nov., a novel water-derived actinobacterium

**DOI:** 10.1371/journal.pone.0340783

**Published:** 2026-02-10

**Authors:** Shuangqing Zhou, Sarah Kirstein, Lara Keunecke, Alexandra Lehmann, Lars-Olaf Schulz, Marlen Jando, Gabriele Pötter, Meina Neumann-Schaal, Yvonne Mast, Imen Nouioui

**Affiliations:** 1 Leibniz-Institute DSMZ – German Collection of Microorganisms and Cell Cultures, Inhoffenstraße 7B, Braunschweig, Germany; 2 College of Pharmacy, Guilin Medical University, Guilin, PR China; 3 Technische Universität Braunschweig, Institut für Mikrobiologie, Rebenring 56, Braunschweig, Germany,; 4 Braunschweig Integrated Centre of Systems Biology (BRICS), Rebenring 56, Braunschweig, Germany; Osmania University, INDIA

## Abstract

Strain DSM 117947^T^ was isolated from a *Micromonospora matsumotoense* co-culture originating from a water sample collected in Germany. The strain was subjected to a polyphasic taxonomic analysis. It exhibited 99.3% 16S rRNA gene sequence similarity with *Nocardioides aurantiacus* DSM 12652^T^. Digital DNA-DNA hybridization and average nucleotide identity values between the strain and its close phylogenetic neighbour were below the threshold of 70% and 95−96% for prokaryotic species demarcation, respectively. The strain had a polar lipid profile composed of diphosphatidylglycerol (DPG), phosphatidylethanolamine (PE), phosphatidylmethylethanolamine (PME), phosphatidylinositol (PI), glycophospholipid (GPL), and phospholipids (PLs). The predominant menaquinone (>20%) was MK-8(H_4_). The major fatty acids (>5%) were C_16.0_, C_16:1_ ω7c, and C_18:1_ ω9c. The genomic G + C content of the strain is 73%. The chemotaxonomic, biochemical, enzymatic, and genomic features distinguished the strain from its close relative, and justify its assignment to a novel species, for which the name *Nocardioides aquaegermanicae* sp. nov., is proposed, with strain DSM 117947^T^ (WG_orange^T^ = KCTC 59414^T^) as the type strain.

## Introduction

The genus *Nocardioides* of the family *Nocardioidaceae* within the order *Propionibacteriales* comprises 171 validly named species (https://lpsn.dsmz.de/ [accessed in July 2025]). Members of this genus have been isolated from different environments, including plastic waste [1], Arctic marine sediments [2], stony coral [3], waterfalls [4], the feces of Tibetan antelope [5,6], kaolinite clay [7], sewage sludge [8], the gastrointestinal tract of lake prawn [9], intestines of fish species [10], and cave stalactite surfaces [11]. *Nocardioides* strains are Gram-positive, aerobic, non-acid-fast bacteria, exhibiting coccoid, short rod-shaped, rod-shaped, or filamentous cell morphologies. Several *Nocardioides* species do not produce aerial mycelium. The cell wall peptidoglycan contains *LL*-diaminopimelic acid as the diagnostic diamino acid. The predominant menaquinone is MK-8 (H_4_), and the major cellular fatty acid is 14-methyl pentadecanoic acid (*iso*-C_16:0_). The average genome size is approximately5.7 Mb, with a DNA G + C contentranging from 67.5% to74.8 mol% [12].

*Nocardioides* strains are known for their ability to utilize various carbon and nitrogen sources, and degrade various organic compounds and environmental pollutants, such as hydrocarbons, haloalkanes, aromatic compounds, nitrogen-containing heterocycles, and polyester pollutants. For example, *Nocardioides* sp. KP7, *Nocardioides* simplex FJ2-1A, *Nocardioides* sp. JQ2195, *Nocardioides carbamazepine, Nocardioides alcanivorans* NGK65^T^, *Nocardioides limicola* DJM-14^T^, and *Nocardioides* sp. JWJ-L0 have been reported to degrade phthalates [13], 2,4-dinitroanisole [14], dibenzofuran [15], ibuprofen [16], hexadecane [17], alkanes [18], and polycyclic aromatic hydrocarbons [19], respectively. In the course of activating and clarifying the taxonomic status of old actinobacterial strains deposited at the DSMZ before the 1990s, several strains belonging to different orders of the phylum *Actinomycetota* were found to be candidates for novel species. In this context, *Nocardioides* sp. DSM 117947^T^ was obtained from a purification of a culture of *Micromonospora matsumotoense* isolated from water collected in Germany and deposited at the DSMZ open culture collection. Given the bioremediation potential of *Nocardioides*, strain DSM 117947^T^ was subjected to a comprehensive polyphasic taxonomic analysis, including comparative genomic studies. The results demonstrated that the strain represents a novel species of the genus *Nocardioides*, for which the name *Nocardioides aquaegermanicae* sp. nov. is proposed.

## Materials and methods

### Origin and maintenance

The strain DSM 117947^T^ (WG_orange^T^ = KCTC 59414^T^) was isolated from a co-culture of *Micromonospora matsumotoense*, which was isolated from water collected in Germany before 1993 and deposited at the DSMZ culture collection. The strain was purified after serial dilution of the original culture. One hundred microliters of bacterial suspension (dilution 10^−6^) dissolved in NaCl solution (0.9% w/v) was spread on GYM medium [glucose (4 g), yeast extract (4 g), malt extract (10.0), CaCO_3_ (2.0), agar 20.0g, H_2_O (1l), pH 7.2] and incubated at 28 °C for 10 days. A single colony was streaked onto GYM medium and incubated at 28 °C for 10 days. The purity of strain DSM 117947^T^ was confirmed using a light microscope (Nikon Eclipse, 100x objective). For comparative analysis, the closest phylogenetic neighbor of the strain, *Nocardioides aurantiacus* DSM 12652^T^, was included in the study. Information on the origin, history, and growth conditions of strain DSM 12652^T^ is available in the DSMZ online catalogue (https://www.dsmz.de/collection/catalogue). Phenotypic characterisation and molecular identification were carried out using wet biomass from a 10-day-old culture grown in ISP2 liquid medium [glucose (4 g), yeast extract (4 g), malt extract (10.0), agar 20.0g, H_2_O (1l), pH 7.2] at 28 °C with shaking at 150 rpm. Chemotaxonomic studies were performed using freeze-dried cells, except for fatty acid and menaquinone analyses, for which specific sample preparation protocols were applied.

### Molecular identification and phylogenetic studies

To determine the taxonomic status of strain DSM 117947^T^ at the species rank, genomic DNA was extracted as described previously [20] and the 16S rRNA gene was amplified by PCR using primers 27F (5′–AGAGTTTGATC(AC)TGGCTCAG–3′) and 1492R (5′–ACGG(CT)TACCTTGTTACGACTT–3′) [21]. Amplicons were sequenced using the Applied Biosystems (ABI) 96-capillary-system, as described by Risdian et al. [22]. The nearly complete 16S rRNA gene sequence (1525 bp) of strain DSM 11794^T^ was compared with those of validly named *Nocardioides* species, using the EzBioCloud server [23]. The pairwise 16S rRNA gene sequence similarity between strain DSM 117947^T^ and its closest phylogenetic neighbour was determined using EzBioCloud server. Maximum-likelihood (ML) [24–25] and Neighbour Joining (NJ) [26] phylogenetic trees were constructed via MEGA software (v 11.0) with 1000 replicates [27].

### Comparative genomic studies

Biomass harvested from strain DSM 117947^T^ culture, grown under the conditions described above, was used for DNA extraction and whole-genome sequencing by MicrobesNG (Birmingham, UK; https://microbesng.com/). The Illumina NovaSeq 6000 (Illumina, San Diego, USA) platform with a 250 bp paired-end protocol was used for sequencing. Library construction, assembly, and quality control were carried out by the service. Genome quality (completeness and contamination) was assessed via CheckM (v1.2.4) [28]. Genome of the strain was automatically annotated by the NCBI GenBank via PGAP (Prokaryotic Genome Annotation Pipeline) (https://www.ncbi.nlm.nih.gov/datasets/genome/GCF_052591715.1/) [29]. The genome sequence was deposited in the DDBJ/ENA/GenBank database under the accession number JBQOIK000000000.

Genome-based phylogeny and digital DNA–DNA hybridization (dDDH) values between strain DSM 117947ᵀ and its closest phylogenomic relatives were carried out using the Type Strain Genome Server (TYGS) (https://tygs.dsmz.de/) [30–32]. Average nucleotide identity (ANI) scores between the strain and its neighbours were calculated using the ANI calculator available in the EzBioCloud server (https://www.ezbiocloud.net/tools/ani) [33].

### Chemotaxonomic properties of strain DSM 117947^T^

Freeze-dried cells of strain DSM 117947^T^ and its close relative *N. aurantiacus* DSM 12652^T^ were analysed, using thin layer chromatography, for whole-cell sugars [34,35], polar lipids [36], and diaminopimelic acid isomers [37]. Isoprenoid quinones of strain DSM 117947^T^ were examined by high-performance liquid chromatography (HPLC) coupled with a diode array detector and high-resolution mass spectrometry (MS) [38]. Cellular fatty acid profiles of the strains were determined using gas chromatography–mass spectrometry (GC-MS) on an Agilent GC-MS 7000D instrument [39]. The position of double bonds in fatty acids was determined by derivatization of fatty acid methyl esters with dimethyl disulfide [40].

### Growth properties, biochemical, and enzymatic features of strain DSM 117947^T^

The growth of strain DSM 117947^T^ was assessed on a variety of media, including DSMZ 1746 (ISP1), DSMZ 987 (ISP2), DSMZ 84 (ISP3), DSMZ 252 (ISP4), DSMZ 993 (ISP5), DSMZ 1269 (ISP6), DSMZ 1619 (ISP7), DSMZ 535 (TSA, Tryptic Soy Agar), DSMZ 65 (Glucose-Yeast-Malt extract, GYM), DSMZ 83 (Czapek’s agar), and DSMZ 554 (N-Z-Amine agar). The composition of these media is available in the cultivation media database (https://mediadive.dsmz.de/). Growth was tested across a range of different temperatures (4, 10, 16, 20, 25, 28, 35, 37, 42, and 45°C), pH (5, 5.5, 6.0,6.5, 7.0,7.5, 8.0,8.5, and 9.0), and salinity (0%, 2.5%, 5%, 7.5%, and 10% NaCl w/v). The following buffer systems were used: 0.1 M citric acid/ 0.1 M sodium citrate for pH < 6; 0.1 M KH_2_PO_4_/0.1 M NaOH for pH 8.0–8.5; and 0.1 M NaHCO_3_/0.1 M Na_2_CO_3_ for pH 9.0. The morphology of the strain including the colour of the substrate mycelium was determined using RAL colour charts [41]. The cell structures from 7- and 14-day-old cultures, prepared on GYM medium at 28 °C, were observed using a lightmicroscope (Nikon Eclipse, 100x objective).

The biochemical and enzymatic profiles of strain DSM 117947ᵀ and its closest phylogenomic neighbour, *N. aurantiacus* DSM 12652^T^ were examined using API 20NE, API 50CH, and API ZYM test strips according to the manufacturer’s instructions (bioMérieux, France). All tests were inoculated with a homogeneous bacterial suspension equivalent to a density of 5 on the McFarland scale, prepared from cultures grown in ISP2 liquid medium for 10 days at 28°C with shaking at 150 rpm.

### Potential applications of strain DSM 117947^T^

The genome sequence of strain DSM 117947^T^ and its close phylogenomic neighbour, *N. aurantiacus* DSM 12652^T^, were screened for secondary metabolite biosynthetic gene clusters (BGCs) using the antiSMASH web tool version 8.0 with default settings (https://antismash.secondarymetabolites.org) [[42]]. Antimicrobial activity of crude extracts from strains DSM 117947^T^ and DSM 12652^T^ was tested against Gram-negative bacteria (*Escherichia coli* Δ*tolC* JW5503–1 and *Proteus vulgaris* DSM 2140), Gram-positive bacteria (multi-resistant *Staphylococcus aureus* DSM 18827 and *Enterococcus faecium* DSM 20477^T^), and yeast (*Candida albicans* DSM 1386). Crude extracts were prepared from 10-day old cultures of the strains grown in 50 ml ISP2, NL19, NL800, and R5 media, respectively, at 28°C with shaking at 180 rpm, as described previously [43]. The potential of strain DSM 117947^T^ for xenobiotic biodegradation and neutralising biotic and abiotic stresses was evaluated using the PGPT-Pred tool, available in PLaBAse server (https://plabase.cs.uni-tuebingen.de/pb/plabase.php). The aligner used for this study is Blast and blastp+hmmer [44–46].

## Results and discussion

### Taxonomic novelty and distinctive genomic features of strain DSM 117947^T^

**Fig 1:**
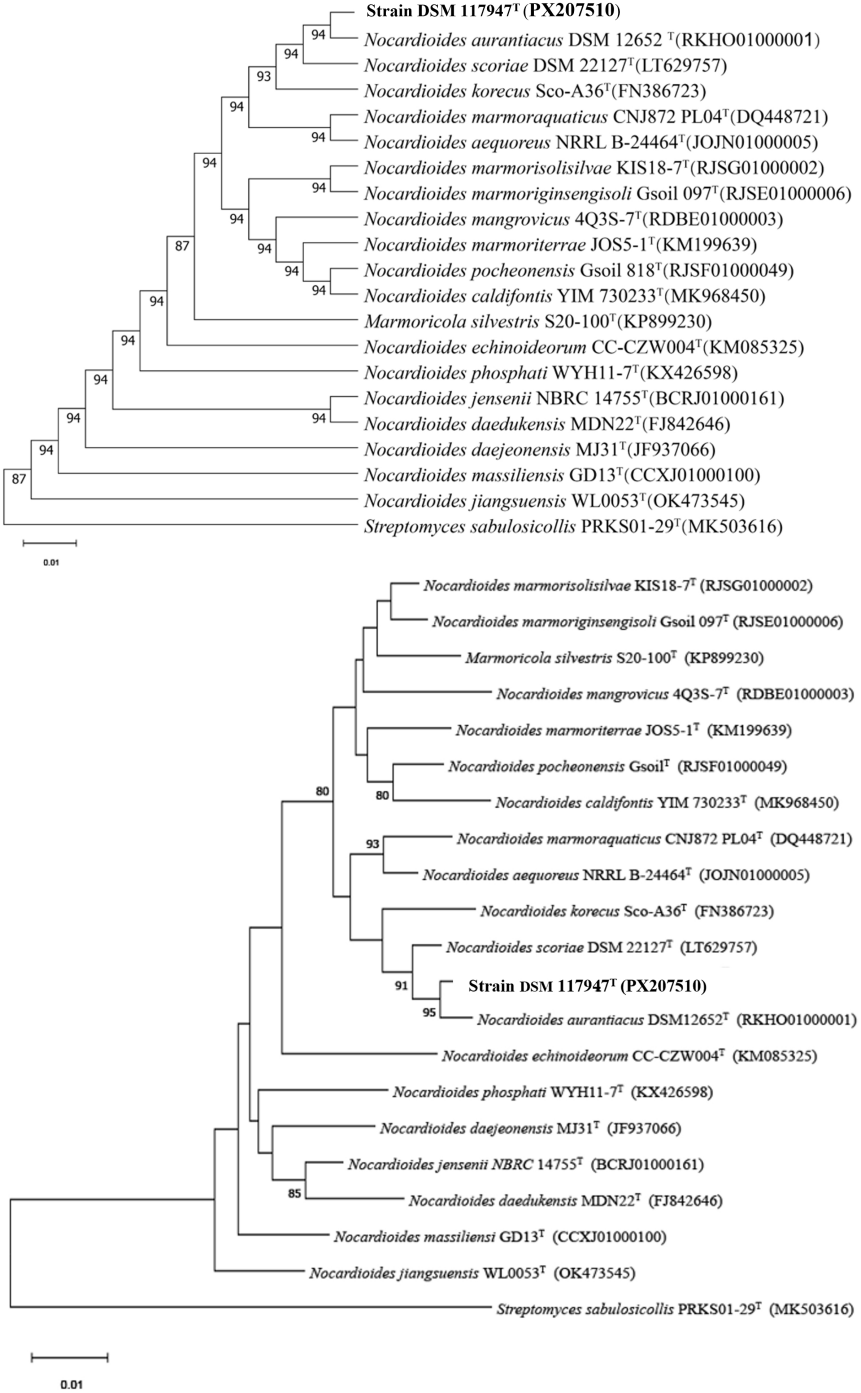
ML and NJ phylogenetic trees based on the 16S rRNA gene sequences showing the phylogenetic position of strain DSM 117947^T^ within the radiation of the genus *Nocardioides.* The numbers above the branches are support values when greater than 50% from ML and NJ bootstrapping. Bar indicates 1 nucleotide substitutions per 1000 nucleotides.

The authenticity of strain DSM 117947^T^ was confirmed by comparing the 16S rRNA gene sequence obtained by PCR with that extracted from the whole genome sequence. The 16S rRNA gene sequence similarity values between strain DSM 117947^**T**^ and validly named *Nocardioides* species ranged from 94.7% to 99.3%, with *N. aurantiacus* (99.3%) identified as the closest relative (S1 Table). These results were consistent with the close phylogenetic relatedness between DSM 117947^T^ and *N. aurantiacus* in the ML and MP phylogenetic trees, in which these strains formed a well-supported sub-cluster next to *Nocardioides scoriae* (Fig 1).

**Fig 2:**
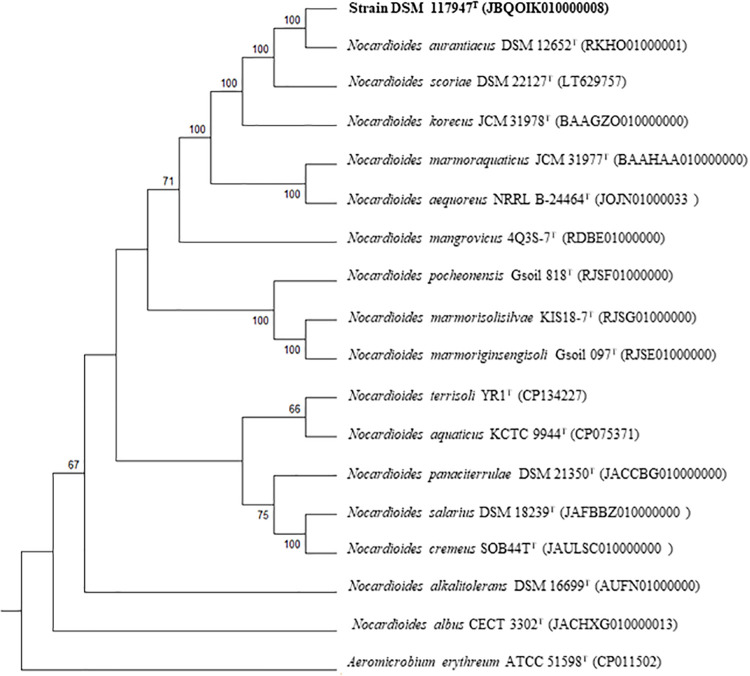
Tree inferred with FastME from GBDP distances calculated from genome sequences. The branch lengths are scaled in terms of GBDP distance formula *d*_*5.*_ Only Bootstrap values above 60% are displayed above the branches.

The genomes of strain DSM 117947^T^ and its close neighbour *N. aurantiacus* DSM 12652^T^ had a size of 4.1 Mbp and 4.0 Mbp, with G + C contents of 73.0% and 72.5%, containing 49 and 54 RNAs, and 4,077 and 3,905 coding sequences, respectively (Table 1). The genomic features of strain DSM 117947^T^ were consistent with those reported for the genus *Nocardioides,* which typically exhibit G + C contents ranging from 67.5 to 74.8% [12]. The whole genome-based tree further confirmed the close phylogenetic relationship between strain DSM 117947ᵀ and *N. aurantiacus* (Fig 2)*.* The dDDH and ANI values between the genome sequences of DSM 117947^T^ and *N. aurantiacus* DSM 12652^T^ were 35.2% and 88.6%, respectively; values well below the accepted species delineation thresholds of 70% for dDDH and 95–96% for ANI [49,50] (S2 Table).

**Table 1:** Biochemical, enzymatic and genomic features that distinguish strain DSM 117947^T^ from its close phylogenetic neighbour *Nocardioides aurantiacus* DSM 12652^T^.

Phenotypic features traits	Strain DSM 117947^T^	Strain DSM 12652^T^	*N. scoriae* DSM 22127^T^**	*N. korecus* JCM 31978^T^***	*N. albus* CECT 3302^T^ ****
**Growth properties**					
pH	5.5-9.0	5.1-8.7*	6.1-12.1	5.1-12.1	6-9
NaCl (%)	0-2.5	0.5-2.0*	0-3.0	0-2.0	0-8
Temperature (°C)	10-37	18-28*	10-37	4-37	15-42
**Utilization as a sole carbon source**					
Glycerol	+	+	–	–	+
D-arabinose	–	+	+	–	+
L-arabinose	+	+	+	ND	+
D-fructose	w	w	+	–	+
D-galactose	+	+	+	–	+
D-mannitol	–	+	+	+	+
Maltose	+	–	+	–	+
D-trehalose	+	+	+	–	–
D-raffinose	+	–	+	–	–
L-rhamnose	–	–	+	–	+
Inositol	–	–	–	–	–
Sucrose	–	+	+	–	+
**Degradation tests**					
Gelatin	+	–	+	+	+
Tyrosine	+	–	–	–	+
Esculin	–	+	+	+	w
**Enzymatic profile**					
Acid phosphatase	+	+	+	+	–
α-Galactosidase	+	–	–	–	–
β-Glucosidase	–	+	+	+	w
ß-galactosidase	–	+	+	–	v
Lipase	–	+	+	–	–
**Chemotaxonomic features**					
Peptidoglycan	*LL*-DAP	*LL*-DAP*	*LL*-DAP	*LL*-DAP	*LL*-DAP, glycine
Whole cell sugar	Galactose, glucose, and ribose	Glucose, ribose	ND	ND	galactose and glucose
Polar lipid profile	PI, PE, PME, DPG, PL, GL	PI, PG, DPG, PL	DPG, PC, PG, PI, PL	PI, DPG, PG, PC, PL	PG, PI, OH-PI, PL APG, PIMs, DPG
Quinone profile	MK-8 (H_4_) (86.1%), MK-8 (H2) (1.3%), MK-7(H_4_) (12.6%)	MK-8(H_4_) (73.0), MK-8 (H2) (1.0%), MK-7(H_4_) (4.0%), MK-6 (H_4_) (1.0%)*	MK-8(H_4_)	MK-8(H_4_)	MK-8(H_4_), MK-8(H2), MK-8
Fatty acids profile (>5%)	C_16.0_, C_16:1_ ω7c, C_18:1_ ω9c	C_16.0_, C_16:1_ ω7c, C_18:1_ ω9c	C_16: 0_, C_18: 1_ ω9c, 10-methyl C_18: 0_	C_16: 0_, C_17: 1_ ω8c, C_18: 1_ ω 9c	C_16:0_ *iso*, C_17:1_ ω6c, C_17:0_ 10-methyl
**Genomic features**					
Genome Size (Mb)	4.2	4.1	4.1	4.3	5.7
Contigs	35	–	1	48	36
N50	459.3 kb	4.1Mb	4.1Mb	462.735kb	266.7 kb
L50	4	1	1	4	7
GC (%)	73.0	72.5	73.5	72.5	68.6
Genes	4035	3979	3871	4316	5349
Protein coding	3949	3905	3787	4240	5289
Genome completeness (%)	97.71	97.73	97.71	ND	98.72
Genome contamination (%)	2.55	1.93	2.09	ND	5.43
GenBank accession numbers	JBQOIK000000000	NZ_RKHO00000000.1	LT629757	BAAGZO010000000	JACHXG010000013

+, positive; -, negative; v, variable; w, weakly positive, ND, not determined. * data taken from Urzì et al. [46], ** data taken from Lee et al.2010 [47], *** data taken from Lee et al. 2011 [48], **** data taken from Evtushenko et al. [12] . DAP, diaminopimelic acid, DPG, diphosphatidylglycerol, GPL, glycophospholipid, OH-PI, hydroxy-phosphatidylinositol, PC, phosphatidylcholine, PE, phosphatidylethanolamine, PG, phosphatidylglycerol, PI, phosphatidylinositol, PIM, phosphatidylinositolmannoside, PLs, phospholipids, PME, phosphatidyl-N-methylethanolamine. Strains DSM 117947^T^ and DSM 12652^T^ were able to use melibiose and produce acid phosphatase, alkaline phosphatase, α-chymotrypsin, cystine arylamidase, α-glucosidase, esterase, esterase lipase, leucine arylamidase, Naphthol-AS-BI-phosphohydrolase, trypsin, and valine arylamidase. Strains DSM 117947^T^ and DSM 12652^T^ were not able to metabolise *myo*-inositol, lactose, D-, L-rhamnose, D-ribose. All strains were able to metabolise D-cellobiose, D-glucose, D-mannose, and D-xylose.

### Growth properties, phenotypic, and chemotaxonomic features of strain DSM 117947^T^

The phenotypic and morphological characteristics of strain DSM 117947^T^ were consistent with those of the genus *Nocardioides* [23]. The strain grew well on ISP1, ISP2, ISP6, TSA, GYM, Czapek’s agar, and N-Z-Amine agar media, forming a yellow substrate mycelium after 10 days of incubation at 28°C (RAL 1028). The morphology of the strain in GYM medium plate is available in the DSMZ online catalogue (https://www.dsmz.de/collection/catalogue/details/culture/DSM-117947). Strain DSM 117947^T^ showed good growth on GYM medium at 16, 20, 25, 28, and 37°C. The strain grew at pH values ranging from 5.5 to 9.0 and tolerated up to 2.5% NaCl in GYM medium. Optimal growth on GYM medium was observed at 28, 35, and 37°C at pH 7.5–8.5, and in the absence of added salt. The strain developed yellow colonies under optimal growth condition (RAL 1028). Coccoid-shaped cells were observed in 15 days-old culture after incubation at 28°C (S1 Fig).

Strain DSM 117947^T^ was able to metabolise L-arabinose, cellobiose, D-fructose, galactose, D-glucose, glycerol, maltose, D-mannose, melibiose, D-raffinose, trehalose, and D-xylose, as sole carbon sources (Table 1). Strain DSM 117947^T^ was distinguished from its close phylogenetic neighbour, strain DSM 12652^T^, by its ability to hydrolyse gelatin, oxidise tyrosine, and produce alkaline phosphatase, α-galactosidase, and lipase (Table 1). In addition, strain DSM 117947^T^ could be distinguished from its phylogenetic relatives, *Nocardioides scoriae* and *Nocardioides korecus,* and the type species, *Nocardioides albus,* by several biochemical and enzymatic features as shown in Table 1.

Whole-cell hydrolysates of strain DSM 117947^T^ were rich in *LL*-diaminopimelic acid (*LL*-DAP) and galactose, glucose, and ribose as whole cell sugars while *N. aurantiacus* DSM 12652ᵀ lacked galactose [51]. The polar lipid profile of strain DSM 117947^T^ consisted of diphosphatidylglycerol (DPG), phosphatidylethanolamine (PE), phosphatidylmethylethanolamine (PME), phosphatidylinositol (PI), glycolipid (GL), and phospholipids (PLs) (S2 Fig), whereas its close relatives *N. aurantiacus*, *N. korecus* and *N. scoriae* lacked PE and contained phosphatidylglycerol (PG) including the type species *N. albus* [12,47,48,51] (Table 1). The menaquinone profile of the strain consisted of MK-8 (H_4_) (89.1%) and MK-7 (H_4_) (12.6%); coherent with its close relatives (Table 1) [12,47,48,51]. The major fatty acids (>5%) of strains DSM 117947ᵀ and DSM 12652^T^,were C_16.0_, C_16:1_ ω7c, and C_18:1_ ω9c (Table 1). C_16.0_ and C_18:1_ ω9c were also present in *N. korecus* and *N. scoriae,* while the type species exhibited a different profile, reflecting its distant phylogenetic relationship to the studied strain (Table 1)*.*

### Potential applications of strain DSM 117947^T^

Genome mining for secondary metabolite BGCs revealed that strain DSM 117947^T^ and its close relative *N. aurantiacus* DSM 12652^T^ each possessed only a single terpene BGC, showing certain similarity to an isorenieratene and carotenoid BGC, respectively (S3 Table). These findings were consistent with the absence of antimicrobial activity of the crude extracts prepared from various production media when tested against different microbial strains, including Gram-positive and Gram-negative bacteria, as well as yeast.

*In silico* genome analysis of strains DSM 117947^T^ and DSM 12652^T^ revealed the presence of genes associated with the degradation of various aromatic compounds, including benzoate, biphenyl, gentisate, p-hydroxybenzoate, quinate, naphthalene, protocatechuate, toluene, salicylate, xylene and (S4 Table). In addition, strain DSM 117947^T^ harboured in its genome several genes whose products are involved in xenobiotics biodegradation, including oil (dichloroethane, vinylchloride, dichloropropene, dichloromethane), and steroid (adrostenedione, epiandrosterone, cholesterol) and styrene (acrylamide, phenylacetate) degradation. Genes associated with heavy metal detoxification (arsenic, bismuth, cadmium, chromate, cobalt, copper, iron, lead, manganese, nickel, selenium, tellurium, and zinc) were also present in the studied genomes. These compounds, which possess diverse toxicological properties, can originate from natural sources or anthropogenic activities, and their accumulation in the environment disrupt ecosystem functions and pose a threat to human health [52,53].

Furthermore, the genomic sequences of strains DSM 117947^T^ and DSM 12652^T^ were rich in genes controlling environmental stresses (heat shock, cold shock, heat inducible, high and low temperature), reflecting their ability to adapt to different ecological habitats and conditions. The strains seemed to possess the genetic machinery necessary to neutralise acidic (soxalate and spermidine), herbicidal (organophosphate and toxoflavin), osmotic (cardiolipin, trigonelline, glycine-betaine metabolism), salinity (magnesium, sodium, proline, riboflavin, pyridoxine, and folate metabolisms), and oxidative (peroxidised compounds, arylpolyene, carotenoids, glutathione, lipoic acid, terpenoids, spermidine and ubiquinone) stresses. Moreover, genes whose products are involved in the resistance to volatile compounds were also detected.

Several microorganisms, including actinobacteria such as *Mycolicibacterium vinylchloridicum*, have been found to mineralize such pollutants and their derivatives [54].These findings reflect the bioremediation potential of the studied strains.

These findings were consistent with the previously reported bioremediation potential of several species of the genus *Nocardioides* [13–19].

This study provides insight into the genetic machinery involved in the catabolism of polycyclic aromatic hydrocarbons. However, further experimental validation is needed to assess the effectiveness with which the proposed novel type strain DSM 117947^T^ degrades environmentally toxic substances.

## Conclusions

Phenotypic, phylogenetic, and genomic data distinguished strain DSM 117947^T^ from all validly named *Nocardioides* species, supporting its classification as a representative of a novel species, for which the name *Nocardioides aquaegermanicae* sp. nov., is proposed.

### Description of *Nocardioides aquaegermanicae* sp. nov

*aquaegermanicae* (a.quae.ger.ma’ni.cae. L. fem. n. aqua, water; L. fem. adj. *germanica*, German; N.L. gen. fem. n. *aquaegermanicae*, of the German water, referring to the freshwater source in Germany from which the type strain was isolated).

Gram-stain-positive, aerobic, catalase positive, oxidase negative with coccoid-shaped cells. The strain exhibits good growth on Czapek’s agar, GYM, ISP1, ISP6, N-Z-Amine agar, and TSA media, producing an orange substrate mycelium. Optimal growth occurs at 28°C, 35°C, and 37°C, within a pH range of 7.5–8.5, and in the absence of salt. The strain utilises L-arabinose, cellobiose, D-fructose, galactose, D-glucose, glycerol, maltose, D-mannose, melibiose, D-raffinose, trehalose, and D-xylose as sole carbon sources. The polar lipids are diphosphatidylglycerol (DPG), phosphatidylethanolamine (PE), phosphatidylmethylethanolamine (PME), phosphatidylinositol (PI), glycolipid (GL), and phospholipids (PLs). The predominant menaquinone (>20%) is MK-8 (H4). The major fatty acids (>5%) are C_16.0_, C_18:1_ ω9c, and C_16:1_ ω7c. The genome size of the type strain DSM 117947^T^ is 4.1 Mbp with a G + C content of 73%.

The type strain DSM 117947^T^ (WG_orange^T^ = KCTC 59414^T^) was isolated from a *Micromonospora matsumotoense* co-culture originating from a water sample collected in Germany. The 16S rRNA gene and whole genome sequences have been deposited at NCBI GenBank under accession numbers PX207510 and JBQOIK000000000, respectively.

This Whole Genome Shotgun project has been deposited at DDBJ/ENA/GenBank under the accession JBQOIK000000000. The version described in this paper is version JBQOIK000000000.

### Repositories

The GenBank accession number for the 16S rRNA gene and genome sequences of strain DSM 117947^T^ are PX207510 and JBQOIK000000000, respectively.

## Supporting information

S1 Fig Morphological features (left) and cell structures (right) of strain 117947^T^ grown on GYM medium at 28 °C for 15 days. The picture on the right was taken using a light microscope (Nikon Eclipse, 100x objective).
(DOCX)

S2 Fig Two-dimensional TLC plate of polar lipids extracted from strain DSM 117947^T^ stained with molybdatophosphoric acid (a), molybdenum blue (b), ninhydrin (c), anisaldehyde (d), Dragendorff (e) reagents. Key: DPG, diphosphatidylglycerol; PE, phosphatidylethanolamine; PI, phosphatidylinositol; PLs, phospholipids; GL glycolipid: PME, phosphatidylmethylethanolamine. Solvent1: chloroform: methanol: distilled water (65:25:4 v/v/v/); solvent 2: chloroform: glacial acetic acid: methanol: distilled water (80:12:15:4 v/v/v).
(DOCX)

S1 Table 16S rRNA gene sequence similarity between strain DSM 117947^T^ and its close phylogenetic neighbours. (DOCX)

S2 Table dDDH and ANI between the genome sequence of strain DSM 117947^T^ and its close phylogenomic relatives. (DOCX)

S3 Table BGCs associated with terpene biosynthesis of strain DSM 117947^T^ and *N. aurantiacus* DSM 12652^T^ assessed using AntiSMASH. (DOCX)

S4 Table Stress envinronmental genes of *Nocardioides* sp. DSM 117947^T^. (XLSX)
