# Supplementary material for: Polyphasic characterization of Nocardioides aquaegermanicae sp. nov., a novel water-derived actinobacterium
Source: PLoS One. 2026 Feb 10;21(2):e0340783. doi: 10.1371/journal.pone.0340783 (PMC12890105; doi:10.1371/journal.pone.0340783)
Supplement: S1 Fig — The picture on the right was taken using a light microscope (Nikon Eclipse, 100x objective). (DOCX) [file pone.0340783.s001.docx]

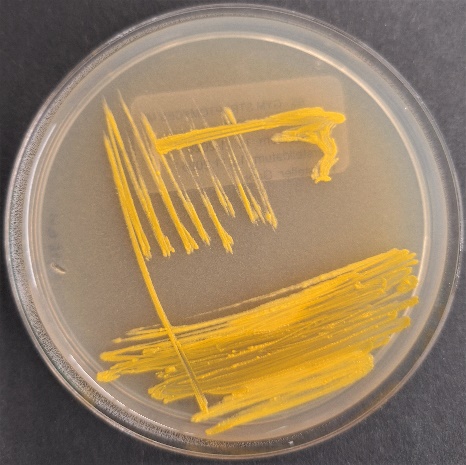

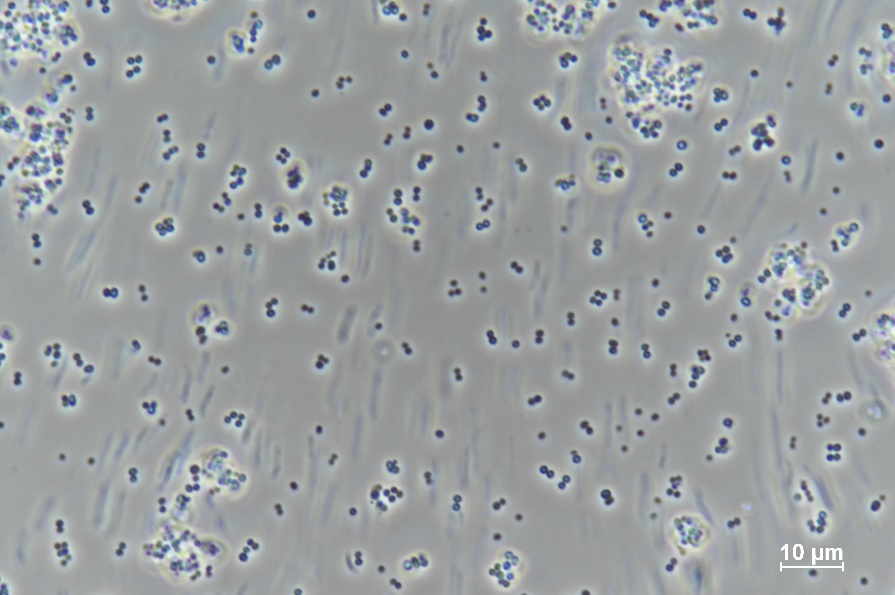


**Figure S1**. Morphological features (left) and cell structures (right) of strain 117947^T^ grown on GYM medium at 28 °C for 15 days. The picture on the right was taken using a light microscope (Nion Eclipse, 100x objective).
