# Supplementary material for: Polyphasic characterization of Nocardioides aquaegermanicae sp. nov., a novel water-derived actinobacterium
Source: PLoS One. 2026 Feb 10;21(2):e0340783. doi: 10.1371/journal.pone.0340783 (PMC12890105; doi:10.1371/journal.pone.0340783)
Supplement: S2 Fig — Key: DPG, diphosphatidylglycerol; PE, phosphatidylethanolamine; PI, phosphatidylinositol; PLs, phospholipids; GL glycolipid: PME, phosphatidylmethylethanolamine. Solvent1: chloroform: methanol: distilled water (65:25:4 v/v/v/); solvent 2: chloroform: glacial acetic acid: methanol: distilled water (80:12:15:4 v/v/v). (DOCX) [file pone.0340783.s002.docx]

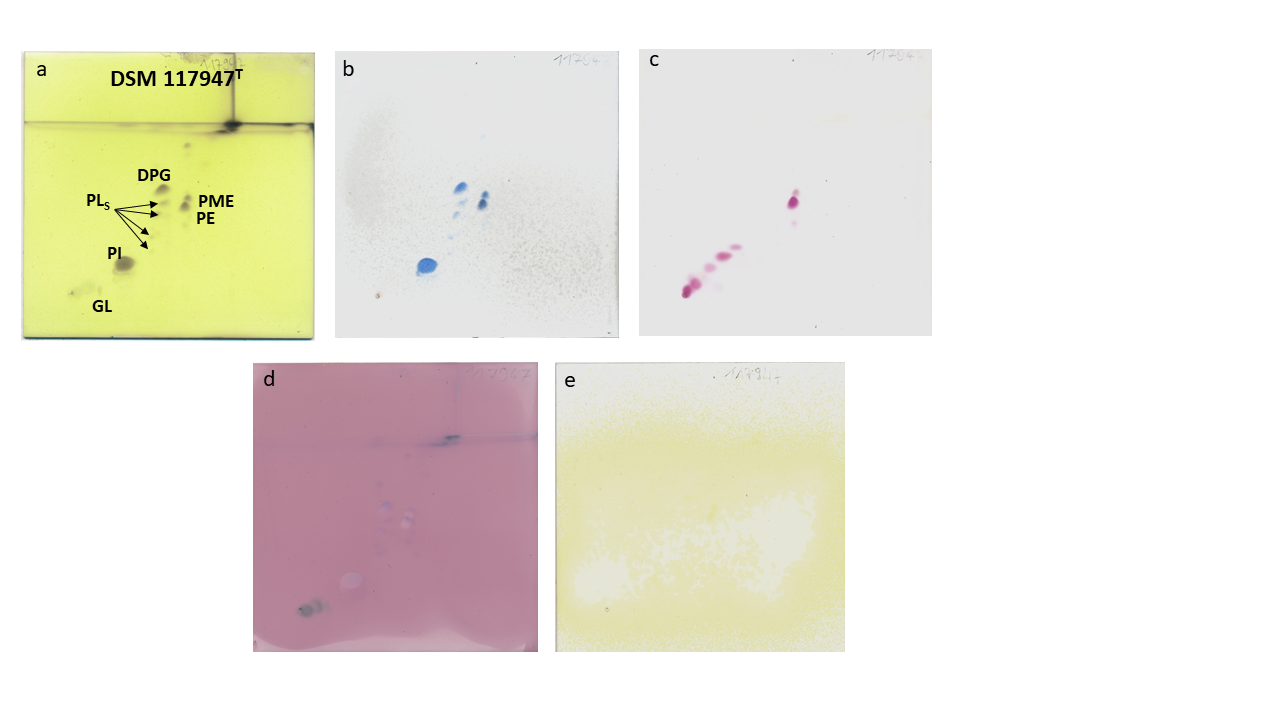


**Figure S2.** Two-dimensional TLC plate of polar lipids extracted from strain DSM 117947^T^ stained with molybdatophosphoric acid (a), molybdenum blue (b), ninhydrin (c), anisaldehyde (d), Dragendorff (e) reagents. Key: DPG, diphosphatidylglycerol; PE, phosphatidylethanolamine; PI, phosphatidylinositol; PLs, phospholipids; GL glycolipid: PME, phosphatidylmethylethanolamine. Solvent1: chloroform: methanol: distilled water (65:25:4 v/v/v/); solvent 2: chloroform: glacial acetic acid: methanol: distilled water (80:12:15:4 v/v/v).
