# Supplementary material for: Polyphasic characterization of Nocardioides aquaegermanicae sp. nov., a novel water-derived actinobacterium
Source: PLoS One. 2026 Feb 10;21(2):e0340783. doi: 10.1371/journal.pone.0340783 (PMC12890105; doi:10.1371/journal.pone.0340783)
Supplement: S1 Table — (DOCX) [file pone.0340783.s003.docx]

**Table S1.** 16S rRNA gene sequence similarity between strain DSM 117947^T^ and its close phylogenetic neighbours.

| Type strains | Accession numbers | 16S rRNA gene sequence similarity (%) |
| --- | --- | --- |
| *Nocardioides aurantiacus* DSM 12652^T^ | RKHO01000001 | 99.31 |
| *Nocardioides scoriae* DSM 22127^T^ | LT629757 | 99.03 |
| *Nocardioides marmorisolisilvae* KIS18-7^T^ | RJSG01000002 | 97.57 |
| *Nocardioides marmoriginsengisoli* Gsoil 097^T^ | RJSE01000006 | 97.50 |
| *Nocardioides korecus* Sco-A36^T^ | FN386723 | 97.46 |
| *Marmoricola silvestris* S20-100^T^ | KP899230 | 97.31 |
| *Nocardioides marmoraquaticus* CNJ872 PL04^T^ | DQ448721 | 97.29 |
| *Nocardioides aequoreus* NRRL B-24464^T^ | JOJN01000005 | 97.15 |
| *Nocardioides pocheonensis* Gsoil 818^T^ | RJSF01000049 | 96.88 |
| *Nocardioides marmoriterrae* JOS5-1^T^ | KM199639 | 96.32 |
| *Nocardioides jensenii* NBRC 14755^T^ | BCRJ01000161 | 96.31 |
| *Nocardioides mangrovicus* 4Q3S-7^T^ | RDBE01000003 | 96.18 |
| *Nocardioides echinoideorum* CC-CZW004^T^ | KM085325 | 96.03 |
| *Nocardioides daedukensis* MDN22^T^ | FJ842646 | 95.90 |
| *Nocardioides caldifontis* YIM 730233^T^ | MK968450 | 95.89 |
| *Nocardioides phosphati* WYH11-7^T^ | KX426598 | 95.83 |
| *Nocardioides daejeonensis* MJ31^T^ | JF937066 | 95.76 |
| *Nocardioides massiliensis* GD13^T^ | CCXJ01000100 | 95.76 |
| *Nocardioides jiangsuensis* WL0053^T^ | OK473545 | 95.72 |
| *Nocardioides cavernaquae* K1W22B-1^T^ | QYRP01000002 | 95.69 |
| *Marmoricola endophyticus* 8BXZ-J1^T^ | KY921599 | 95.62 |
| *Nocardioides campestrisoli* MC1495^T^ | MT476852 | 95.62 |
| *Nocardioides marmoribigeumensis* MSL-05^T^ | EF466120 | 95.56 |
| *Nocardioides pakistanensis* NCCP-1340^T^ | LC065367 | 95.55 |
| *Nocardioides malaquae* Y6^T^ | MW019669 | 95.48 |
| *Nocardioides donggukensis* MJB4^T^ | MN900647 | 95.48 |
| *Nocardioides dubius* KSL-104^T^ | AY928902 | 95.34 |
| *Nocardioides alcanivorans* NGK65^T^ | OU764944 | 95.34 |
| *Nocardioides mesophilus* MSL-22^T^ | EF466117 | 95.34 |
| *Nocardioides solisilvae* Ka25^T^ | LN555580 | 95.32 |
| *Nocardioides iriomotensis* IR27-S3^T^ | AB544079 | 95.29 |
| *Nocardioides pacificus* XH274^T^ | KC986355 | 95.20 |
| *Nocardioides ochotonae* ZJ1313^T^ | MN796260 | 95.13 |
| *Nocardioides guangzhouensis* 130^T^ | MK112565 | 95.13 |
| *Nocardioides jishulii* dk3136^T^ | MK215795 | 95.13 |
| *Nocardioides allogilvus* CFH 30205^T^ | MG800321 | 95.13 |
| *Nocardioides houyundeii* 78^T^ | MG209819 | 95.06 |
| *Nocardioides panacis* G188^T^ | MK796051 | 94.99 |
| *Nocardioides sediminis* MSL-01^T^ | EF466110 | 94.99 |
| *Nocardioides conyzicola* HWE 2-02^T^ | KC878445 | 94.76 |
