# Supplementary material for: Polyphasic characterization of Nocardioides aquaegermanicae sp. nov., a novel water-derived actinobacterium
Source: PLoS One. 2026 Feb 10;21(2):e0340783. doi: 10.1371/journal.pone.0340783 (PMC12890105; doi:10.1371/journal.pone.0340783)
Supplement: S2 Table — (DOCX) [file pone.0340783.s004.docx]

**Table S2**. dDDH and ANI between the genome sequence of strain DSM 117947^T^ and its close phylogenomic relatives.

| **Subject strain** | **Genome Accession** | **dDDH  ( %)** | **ANI** | **G+C content difference  (%)** |
| --- | --- | --- | --- | --- |
| *Nocardioides aurantiacus* DSM 12652^T^ | GCA_003752505 | 65.9 | 88.67 | 0.32 |
| *Nocardioides scoriae* DSM 22127^T^ | GCA_900104965 | 50.5 | 82.90 | 0.47 |
| *Nocardioides aequoreus* NRRL B-24464^T^ | GCF_000720335 | 31.8 | 79.36 | 0.1 |
| *Nocardioides panaciterrulae* DSM 21350^T^ | GCA_013409645 | 19.3 | 82.90 | 0.23 |
| *Nocardioides aquaticus* KCTC 9944^T^ | GCA_018459925 | 20.3 | 76.95 | 0.12 |
| *Nocardioides cremeus* SOB44^T^ | GCA_030518195 | 20.0 | 76.49 | 0.49 |
| *Nocardioides pocheonensis* Gsoil 818^T^ | GCA_003725535 | 18.6 | 76.81 | 2.03 |
| *Nocardioides mangrovicus* 4Q3S-7^T^ | GCA_003674065 | 19.3 | 76.44 | 1.06 |
| *Nocardioides marinisabuli* DSM 18965^T^ | GCA_013409965 | 20.0 | 76.51 | 0.14 |
| *Nocardioides salarius* DSM 18239^T^ | GCA_016907435 | 20.0 | 76.45 | 0.22 |
| *Nocardioides marmorisolisilvae* KIS18-7^T^ | GCA_003725775 | 18.9 | 76.61 | 3.4 |
| *Nocardioides terrisoli* YR1^T^ | GCA_031656915 | 17.6 | 75.93 | 3.36 |
| *Nocardioides marmoriginsengisoli* Gsoil 097^T^ | GCA_003725695 | 18.1 | 76.55 | 2.8 |
| *Nocardioides sediminis* KCTC 19271^T^ | GCA_003047295 | 19.3 | 76.40 | 0.72 |
| *Nocardioides jensenii* NBRC 14755^T^ | GCA_001552535 | 16.4 | 75.34 | 4.11 |
| *Nocardioides alkalitolerans* DSM 16699^T^ | GCA_000426525 | 17.5 | 75.37 | 0.05 |
| *Nocardioides albus* CECT 3302^T^ | GCF_014191915 | 15.4 | 74.09 | 4.09 |
| *Nocardioides caldifontis* YIM 730233^T^ | GCA_008087345 | 17.7 | 75.49 | 0.9 |
| *Aeromicrobium erythreum* ATCC 51598^T^ | GCA_001509405 | 15.1 | 72.96 | 0.89 |
