# Supplementary material for: Polyphasic characterization of Nocardioides aquaegermanicae sp. nov., a novel water-derived actinobacterium
Source: PLoS One. 2026 Feb 10;21(2):e0340783. doi: 10.1371/journal.pone.0340783 (PMC12890105; doi:10.1371/journal.pone.0340783)
Supplement: S3 Table — (DOCX) [file pone.0340783.s005.docx]

**Table S3**. BGCs associated with terpene biosynthesis of strain DSM 117947^T^ and *N. aurantiacus* DSM 12652^T^ assessed using AntiSMASH.

| **Type** | **Most similar known cluster** | **DSM 117947**^T^ | **DSM 12652**^T^ |
| --- | --- | --- | --- |
| Terpene | Isorenieratene | medium | - |
| Terpene | carotenoid | - | low |

-, not present; medium and low are for cluster similarity between 75% - 50% and 50% - 15%, respectively (Blin et al. 2025)
